# An Intronic HCP5 Variant Is Associated With Age of Onset and Susceptibility to Graves Disease in UK and Polish Cohorts

**DOI:** 10.1210/clinem/dgaa347

**Published:** 2020-06-05

**Authors:** Laura Claire Lane, Aleksander Kuś, Tomasz Bednarczuk, Artur Bossowski, Jacek Daroszewski, Beata Jurecka-Lubieniecka, Heather Jane Cordell, Simon Henry Schofield Pearce, Timothy Cheetham, Anna Louise Mitchell

**Affiliations:** 1 Translational and Clinical Research Institute, Newcastle University, Newcastle-upon-Tyne, UK; 2 Endocrine Unit, Royal Victoria Infirmary, Newcastle-upon-Tyne, UK; 3 Department of Paediatric Endocrinology, The Great North Children’s Hospital, Newcastle-upon-Tyne, UK; 4 Department of Internal Medicine and Endocrinology, Medical University of Warsaw, Warsaw, Poland; 5 Department of Pediatrics, Endocrinology and Diabetes with a Cardiology Unit, Medical University of Bialystok, Bialystok, Poland; 6 Department of Endocrinology, Diabetes and Isotope Therapy, Wroclaw Medical University, Wroclaw, Poland; 7 Department of Nuclear Medicine and Endocrine Oncology, Maria Sklodowska-Curie Institute - Oncology Center, Gliwice Branch, Gliwice, Poland; 8 Population Health Sciences Institute, Newcastle University, Newcastle-upon-Tyne, UK

**Keywords:** thyroid, autoimmune, polymorphism, genotyping, Graves disease, meta-analysis

## Abstract

**Context:**

The genetic background of young-onset Graves disease (GD) remains largely unknown. An intronic variant in human leukocyte antigen (HLA) complex P5 (HCP5) has previously been associated with GD susceptibility and age of onset in a cohort of Polish patients.

**Objective:**

We aimed to investigate the association of the *HCP5* variant *rs3094228* with GD susceptibility and age of onset in a UK cohort and conduct a meta-analysis of UK and Polish data.

**Design and Participants:**

*rs3094228* was genotyped in 469 UK patients with GD using Taqman chemistry. Genotype frequencies were compared with genotypic data available from the Wellcome Trust case-control consortium using logistic regression analysis. To determine whether *rs3094228* is independently associated with age of GD onset, the HLA DRB1*0301 tagging variant, *rs535777,* was also genotyped.

**Results:**

The C allele of *rs3094228* was overrepresented in the UK GD cohort compared with controls (P ^allele^=5.08 × 10^–9^, odds ratio 1.76; [95% confidence interval, 1.46-2.13]). This association was more marked in young-onset GD (<30 years) (P ^allele^=1.70 × 10^–10^ vs P ^allele^=0.0008). The meta-analysis of UK and Polish data supported the association of the C allele with GD susceptibility (P ^allele^=1.79 × 10^–5^) and age of onset (P ^allele^=5.63 × 10^–8^). Haplotype analysis demonstrated that *rs3094228* is associated with age of GD onset (*P* = 2.39 × 10^-6^) independent of linkage disequilibrium with HLA DRB1*0301.

**Conclusion:**

The *rs3094228 HCP5* polymorphism is independently associated with GD susceptibility and age of onset in a UK GD cohort. Our findings indicate a potential role of long noncoding ribonucleic acids, including *HCP5,* in GD pathogenesis, particularly in the younger population.

Graves disease (GD) is the most common cause of hyperthyroidism in young children and adolescents; however, it is still relatively rare, with a reported incidence of between 0.1 and 3 per 100 000 (1) compared with Caucasian European adults where the incidence is reported as 20 to 25 cases per 100 000 (2, 3). Longitudinal studies have reported an increasing incidence of hyperthyroidism in both adult and pediatric populations (4-6). Similar to other autoimmune conditions, there is a clear female preponderance, with GD affecting up to 3% of women and 0.5% of men, with a peak incidence occurring between 30 and 50 years of age (2).

GD is characterized by the presence of thyroid receptor autoantibodies (TRAbs) that stimulate the cell-surface thyroid-stimulating hormone (TSH) receptor, directly resulting in excessive, autonomous thyroid hormone secretion. The clinical features and prognosis of GD is highly variable depending on age of disease onset, with the remission rate following a course of antithyroid medication being as low as 25% in the pediatric population compared with 50% to 60% in adults (7, 8).

GD results from a complex interaction between genetic and environmental factors involving variants in multiple susceptibility genes, each exerting modest individual effects. Family and twin studies over the past 50 years have demonstrated that up to 80% of an individual’s predisposition to GD is attributable to genetic factors (9, 10). However, only around 20% of this genetic load has been accounted for by the currently associated genomic variants (11).

Genomic polymorphisms associated with susceptibility to GD are primarily found at immune-regulatory loci, such as *MHC* (12), *CTLA-4* (13), *PTPN22* (14), and *CD40* (15). A stronger genetic association is suspected in the younger population who have had less exposure to environmental factors. Several of the known susceptibility loci are also associated with a younger age of disease onset, including those at *CTLA-4* (16), human leukocyte (*HLA)-DRB1* (17), and *PTPN22* (18), with the most strongly associated variants located at the major histocompatibility complex (MHC) locus (19, 20). Determining genetic variants associated with GD can provide mechanistic insight by highlighting pathogenic functional pathways, particularly by studying the younger population where genetics may be the dominant factor (19).

This study aimed to investigate the association of the *HLA complex P5* (*HCP5*) gene in GD susceptibility and age of onset in a UK cohort. The association between *HCP5* variants and thyroid autoimmunity was first demonstrated in a multicenter population-based genome-wide association study conducted by Medici et al for serum levels of thyroid peroxidase antibodies (21). The first study showing an association of *HCP5* with susceptibility to GD (*rs3094228*, *P* = 1.6 × 10^–12^; odds ratio [OR] = 1.88), was performed in a single center by Kuś et al (22). A subsequent multicenter study with a relatively large pediatric GD cohort demonstrated the *HCP5* variant, *rs3094228*, as a risk locus for young-onset GD (YOGD) (23).

We have studied the same *HCP5* polymorphism in a UK GD cohort and performed a meta-analysis of data from the UK and Polish patient cohorts.

## Materials and Methods

### Participants

A total of 469 patients were included in the UK cohort, including 118 patients with YOGD (aged <30 years) and 351 patients with unrelated later-onset GD (LOGD) (aged ≥30 years). The YOGD cohort included 18 (15%) male and 100 (85%) female (GD onset aged 3-29 years; median 22 years, mean 20.8 years) and the LOGD cohort included 55 (16%) male and 296 (84%) female (GD onset aged 30-92 years; median 47 years, mean 48.2 years).

The patients providing these samples were of Caucasian European background and had attended outpatient endocrinology at the Royal Victoria Infirmary or the Great North Children’s Hospital, Newcastle-upon-Tyne, UK. Each participant with GD was diagnosed by the following criteria: fully suppressed serum TSH with serum free thyroxine and/or free triiodothyronine above the reference range and the existence of detectable TSH receptor antibody (TRAb; ≥1.8 mU/L; Brahms Kryptor).

Genotype data from 5377 control samples from the Wellcome Trust case-control consortium (WTCCC2) database were used for comparison. Informed, written consent was obtained from all participants. This study was carried out with approval of the Leeds East (Ref. 05/Q1206/144) and Berkshire Valley ethics committees (Ref. 04/12/015).

### HCP5 genotyping

The *HCP5* variant *rs3094228* was genotyped in genomic deoxyribonucleic acid extracted from venous blood using TaqMan chemistry as per the manufacturer’s instructions (assay C_2995657_10) and run on the QuantStudio 7 Flex Real-Time PCR (polymerase chain reaction) System (Applied Biosystems). Twenty percent of the samples were genotyped in duplicate to ensure assay fidelity. The overall genotyping call rate was 99.8%.

### HLA genotyping

The HLA DRB1*0301 tagging variant, *rs535777*, was genotyped in the UK cohort as above (assay C__26546461_30). The overall genotyping call rate was 99.6%.

In the Polish cohort, the *HLA-DRB1* polymorphism was genotyped using the low-resolution single specific primer-polymerase chain reaction (SSP-PCR) method with use of the Dynal All Set SSP DR Kit or the HLA-Ready Gene DR Kit, as previously described (22).

### Statistical analysis

Statistical association analysis was performed using PLINK (24) and SPSS version 25 (25). All the control sample genotypes were in Hardy-Weinberg equilibrium (*P* > 0.4). Study data were compared with WTCCC2 control data using logistic regression with sex as a covariate. A subgroup association analysis was performed comparing young-onset (aged <30 years) GD to older-onset (aged ≥ 30 years) GD. A meta-analysis, using the Review Manager (RevMan) Version 5.0 program (Nordic Cochrane Centre, Copenhagen, Denmark (26)) was then undertaken, using a random effects model to calculate ORs, 95% confidence intervals (CI) and 2-sided *P* values. The impact of heterogeneity between the cohorts was estimated using an I^2^ index. Kaplan-Meier plots and log-rank tests were applied to determine whether genotype was significantly associated with age of GD onset. Logistic regression and haplotype analysis (UNPHASED 3.1.7 (27)) was performed to determine the independent association of *rs3094228* with age of GD onset.

## Results

### GD susceptibility

The minor C allele and the CC genotype at *rs3094228* are associated with susceptibility to GD. The frequency of the C allele was significantly increased in the GD cohort as a whole (303/938; 32%) compared with WTCCC2 controls (2118/10 754; 20%: *P* = 5.08 × 10^–9^; OR 1.76 [95% CI, 1.46-2.13]). There was also a significant increase in the CC genotype in the GD group (39/469; 8%) compared with WTCCC2 controls (219/5377; 4%: *P* = 2.89 × 10^–18^) (Table 1).

**Table 1.** Logistic regression analysis with sex as a covariate. Genotype and allele frequencies at *rs3094228* in the GD UK cohort and healthy Controls (WTCCC2), subdivided into age of disease onset (aged < 30 years / ≥ 30 years)

*rs3094228*		WTCCC Controls (%)	All GD UK Cohort (%)	GD Aged < 30 Years (%)	GD Aged ≥ 30 Years (%)
**Genotype frequency**	CC	219 (4)	39 (8)	16 (13)	23 (7)
	CT	1680 (31)	225 (48)	67 (57)	158 (45)
	TT	3478 (65)	205 (44)	35 (30)	170 (48)
*P* value			**2.89 × 10** ^**–18**^	**1.90 × 10** ^**–13**^	**6.97 × 10** ^**–9**^
**Allele frequency**	C	2118 (20)	303 (32)	99 (42)	204 (29)
	T	8636 (80)	635 (68)	137 (58)	498 (71)
*P* value (OR [95% CI])			**5.08 × 10** ^**–9**^ **(OR 1.76 [1.46-2.13])**	**1.70 × 10** ^**-10**^ **(OR 2.73 [2.00-3.71])**	**0.0008** **(OR 1.49 [1.18-1.88])**

Abbreviations: CI, confidence interval; GD, Graves disease; OR, odds ratio; WTCCC, Wellcome Trust case-control consortium.

### GD age of onset

Although an increased frequency of the C allele was present in both the YOGD (99/236; 42%) and LOGD groups (204/702; 29%) compared with controls (2118/10 754; 20%), the difference was more significant in the YOGD group (*P* = 1.70 × 10^–10^; OR 2.73 [95% CI, 2.00-3.71]) compared with the LOGD cohort (*P* = 0.0008; OR 1.49 [95% CI, 1.18-1.88]). In addition, a significant increase in the frequency of the C allele (99/236; 42% vs 204/702; 29%: *P*_allele_=0.00025; OR 1.76 [95% CI, 1.3-2.4]) and CC genotype (16/118; 13% vs 23/351; 7%: *P*_genotype_=0.00059) was observed in the YOGD group when compared with the LOGD cohort (Table 2). This suggests that the C allele and CC genotype have a stronger association with susceptibility to GD at a younger age.

**Table 2.** Association analysis of sex-matched young-onset (aged < 30 years) GD compared with later-onset (aged ≥ 30 years) GD

*rs3094228*		GD Aged < 30 Years (%)	GD Aged ≥ 30 Years (%)
**Genotype frequency**	CC	16 (13)	23 (7)
	CT	67 (57)	158 (45)
	TT	35 (30)	170 (48)
*P* value			**0.00059**
**Allele frequency**	C	99 (42)	204 (29)
	T	137 (58)	498 (71)
*P* value (OR [95% CI])			**0.00025** **(OR 1.76 [1.3-2.4])**

Abbreviations: CI, confidence interval; GD, Graves disease; OR, odds ratio.

### Meta-analysis

A meta-analysis was undertaken using additional genotype data provided by Kuś et al (22) from a study that examined genetic risk loci including *rs3094228* in a Polish GD cohort. The YOGD Polish cohort (aged <30 years) included 66 (19%) male and 280 (81%) female (GD onset aged 3-29 years; median 18 years, mean 19 years), and the LOGD Polish cohort (aged ≥ 30 years) included 194 (22%) male and 672 (78%) female (GD onset aged 30-81 years; median 47 years, mean 48 years).

**Figure 1.**
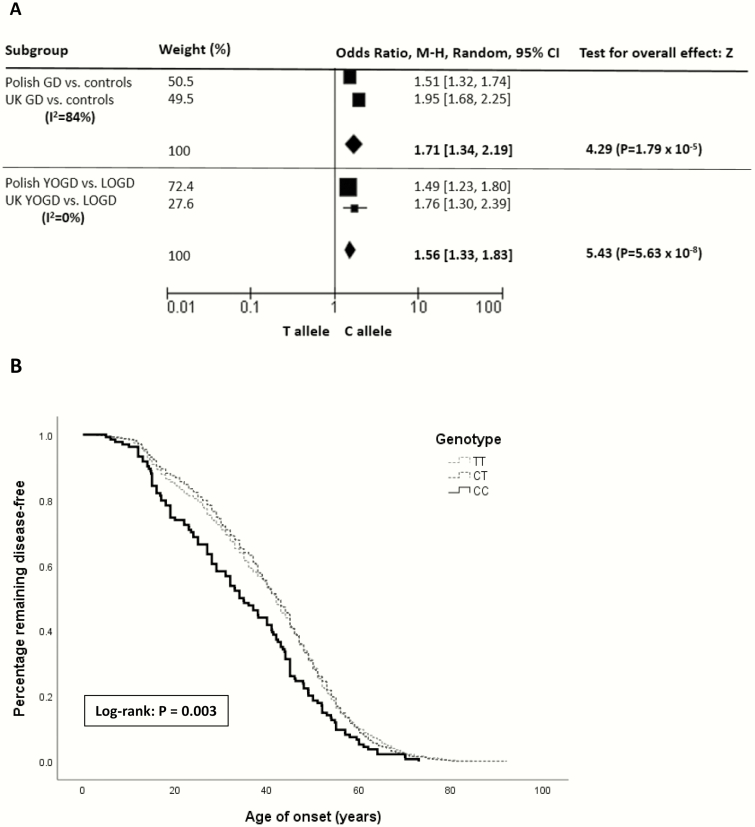
Age-associated cumulative incidence of Graves disease (GD) onset associated with the rs3094228 polymorphism. **(A)** Forest plot of meta-analysis results in GD susceptibility and age of onset in UK and Polish cohorts: meta-analysis using a random effects model of *rs3094228* in the *HCP5* gene examining GD susceptibility compared with controls, and YOGD compared with LOGD. **(B)** Kaplan-Meier analysis and log-rank test. The CC genotype was associated with earlier-onset GD. CI, confidence interval; LOGD, later-onset Graves disease (aged ≥30 years); YOGD, young-onset Graves disease (aged <30 years). Abbreviation: M-H, Mantel-Haenszel test.

Using a random effects model, the association of the C allele with susceptibility to GD (*P* = 1.79 × 10^–5^; OR 1.71 [95% CI, 1.34-2.19]) and an earlier age of disease onset YOGD versus LOGD (*P* = 5.63 × 10^–8^; OR 1.56 [95% CI, 1.33-1.83]) was confirmed (Fig. 1A).

Using the combined UK and Polish cohorts, Kaplan-Meier plots and log-rank tests demonstrated a significant association with genotype and age of GD onset, confirming the association of the CC genotype with earlier-onset GD (*P* = 0.003) (Fig. 1B). In addition, a recessive model demonstrated an increased risk of earlier-onset GD in homozygotes for the minor allele compared with carriers for the common allele (*P* = 0.001). The median age of GD onset in those with the CC genotype was 34 years compared with 40 and 43 years with the CT and TT genotypes, respectively.

### Haplotype analysis

To determine whether the effect of *rs3094228* on age of GD onset is independent of the common GD susceptibility locus, HLA DRB1*0301, a gender-adjusted logistic regression analysis on the Polish data was undertaken, including 439 patients with GD with available data on HLA DRB1*03, *rs3094228*, and age of GD onset. This demonstrated that the observed effect of *rs3094228* on age of GD onset is independent from HLA DRB1*03 (*P* = 0.006).

This was further studied in the UK GD cohort using a known tagging single nucleotide polymorphism for HLA DRB1*0301 in the Northern and Western European (CEU) population (*rs535777*; r^2^=0.87, D’=0.99) (28). Consistent with the Polish data, a gender-adjusted logistic regression analysis on the UK cohort demonstrated that the effect of *rs3094228* on age of GD onset occurs independently of HLA DRB1*0301 (*P* = 0.004). A combined logistic regression analysis including the Polish and UK cohorts (906 patients) demonstrated that the interaction of *HCP5* and HLA DRB1*0301 was significantly associated with age of GD onset (*P* = 0.046) (Table 3). Haplotype analysis with *HCP5* as the test marker and HLA DRB1*0301 as the conditioning marker demonstrated that *HCP5* is independently associated with age of GD onset (*P* = 2.39 × 10^-6^).

**Table 3.** Logistic regression analysis of *rs3094228* and HLA DRB1*03, using the combined UK and Polish cohorts

Polymorphism	B	SE	*P* Value	OR (95% CI)
*rs3094228*	0.330	0.151	**0.028**	1.39 (1.04-1.87)
HLA DRB1*03 / *rs535777*	–0.497	0.200	**0.013**	0.61 (0.41-0.90)
*rs3094228 +* HLA DRB1*03 / *rs535777*	0.389	0.195	**0.046**	1.48 (1.01-2.17)

*rs535777* was used as tagging variant for the HLA DRB1*0301 locus in the UK cohort.

Abbreviations: B, unstandardized regression coefficient; CI, confidence interval; OR, odds ratio; SE, standard error.

## Discussion

This meta-analysis includes more than 1600 participants with GD, including more than 460 early-onset cases, from UK and Polish populations. Given the relative rarity of GD in the pediatric population, combining genotype data sets is essential to improve study power. Despite the established strong hereditary component of GD, particularly in the development of GD at a younger age, there is limited data documenting genetic risk variants specifically accounting for YOGD (19, 29, 30). This study finds a robust association between susceptibility to GD and an earlier age of disease onset with an intronic *HCP5* polymorphism in a UK cohort, replicating previous findings in a Polish population (23).

The *HCP5* ribonucleic acid (RNA) gene is located within the MHC class I region, centromeric of the *HLA-B* gene between the *MICA* and *MICB* genes, and it encodes a long noncoding RNA (lncRNA) (31). *MHC* genes encode cell-surface antigen-presenting proteins that are essential to mount an autoimmune response. Genome-wide association studies have identified polymorphisms at the *MHC* locus as risk variants for many autoimmune and inflammatory diseases, including GD in both Asian and European populations (32, 33). Furthermore, Immunochip genetic analysis has demonstrated that variants within the *MHC* have the strongest association with YOGD (aged <30 years) (19).

The role of the MHC class II region is well established in GD, where antigen presentation of thyroid-stimulating hormone receptor to CD4 + T cells is crucial to drive the B cells to produce the pathogenic TRAb autoantibodies (34). However, genetic variants in the MHC I region (*HLA-B* and *HLA-C*) have also previously been independently associated with GD (35). Indeed, messenger RNAseq analysis of GD thyroid tissue found that *HLA-C* in the MHC I region was the most overexpressed gene compared with controls (36). The primary function of MHC I molecules is to present nonself antigens derived from intracellular sources, such as viruses, to CD8 + cytotoxic T cells, which then mount a cytotoxic response against the presented antigen. It has been proposed that viruses may trigger an autoimmune response through molecular mimicry where viral antigens are structurally similar to self-antigens, or bystander activation where the viral infection triggers a nonspecific activation of autoreactive cells (37). Various *HCP5* variants are demonstrated to promote susceptibility to adverse immune-related cutaneous drug reactions, such as Stevens-Johnson syndrome, as well as being associated with disease progression and viral load in untreated patients with HIV, suggesting a possible specific role for *HCP5* in modifying the immune response to medications or viral infections (38, 39). Certain genetic variants within the MHC I region, such as *HCP5* polymorphisms, may predispose to GD by modifying an individual’s response to an infectious agent, resulting in an excessive immune response with the potential to initiate an autoimmune reaction in genetically susceptible individuals.

The *HCP5* polymorphism *rs3094228* investigated in this study has previously been associated with susceptibility to GD in a Polish cohort (22), and an association has been demonstrated between the C allele and thyroid peroxidase antibody levels in autoimmune thyroid disease (21). Other *HCP5* variants have been associated with susceptibility and autoantibody production in various autoimmune disorders, including systemic lupus erythematous (40), Sjögren syndrome (41), psoriasis, and psoriatic arthritis (42).

The *MHC* gene region is highly polymorphic and characterized by extended linkage disequilibrium (LD), making it challenging to determine functional variants from tagging single nucleotide polymorphisms, which highlights the importance of rigorous case-control matching in genetic association studies. LD analysis indicates that the known GD risk locus, HLA DRB1*03, and *rs3099844* studied in systemic lupus erythematous and Sjögren syndrome, are in partial LD with *rs3094228* (HLA DRB1*03: r^2^ =0.45, D’ =0.86 in the Polish population (22), *rs3099844:* r^2^ =0.69, D’ =0.93 in the British population (28)). However, our haplotype analysis with HLA DRB1*03 demonstrates that *HCP5* is independently associated with age of GD onset.

Interestingly, there is accumulating evidence suggesting that lncRNA, such as HCP5, has a crucial role in the development of autoimmunity by altering the adaptive and innate immune response through transcriptional and epigenetic regulation (32, 43-45). Studies have demonstrated that lncRNAs are associated specifically with autoimmune thyroid disease (AITD), including GD. Indeed, it has been proposed that *rs3094228* alters the expression of thyrocyte ligands (*MICA*, *MICB* and *HLA-C)* of immunoreceptors on natural killer cells in those with AITD, promoting antibody-dependent natural killer cell-mediated cytotoxicity of the thyrocyte (46), making it a potentially functional variant. Furthermore, a study by Shirasawa et al demonstrated that a polymorphism in the promoter region of a B-cell-specific antisense RNA transcript, *SAS-ZFAT*, is associated with susceptibility to AITD (44), and the lncRNA transcript, *Heg*, has demonstrated negative correlation with TRAb concentrations in untreated patients with GD (47). Although the precise mechanism remains to be determined, this suggests that both variation and dysregulation of lncRNAs are implicated in AITD, including GD.

HCP5 is expressed at high levels in cells of the immune system such as the spleen, blood, and thymus (48). The aging immune system may contribute to the phenotypic differences observed between YOGD and LOGD, where involution of the thymus and reduced B- and T-lymphocyte production may alter the mechanisms driving the autoimmune response in the older population (49). Phenotypic differences may also be explained by genetic variation including those in the *MHC* region (HLA subtypes DB1*02, DQA1*05, and DRB1*03), which have been associated with a higher risk of relapse in GD (50).

As this study was performed in a Caucasian population, further studies are required to investigate whether a similar effect is also detected in other (non-Caucasian) populations. Further functional studies should also aim to elucidate the underlying mechanism behind the observed association.

## Conclusion

This study has confirmed a significant association of the *HCP5* polymorphism, *rs3094228*, with GD susceptibility and age of disease onset in a UK cohort and replicates the findings from a study of patients with GD in Poland. Adult-onset and young-onset GD share multiple common genetic risk variants, many of which remain unknown. Our findings indicate a potential role for *HCP5* as a contributor to GD susceptibility, particularly in the younger population. Further research to determine the role of lncRNAs, including *HCP5,* in the pathogenesis of early-onset GD is now warranted.

## Abbreviations

AITD: autoimmune thyroid disease
CI: confidence interval
GD: Graves disease
HCP5: HLA complex P5
HLA: human leukocyte antigen
LD: linkage disequilibrium
lncRNA: long noncoding RNA
LOGD: later-onset GD
MHC: major histocompatibility complex
OR: odds ratio
PCR: polymerase chain reaction
RNA: ribonucleic acid
TRAb: thyroid receptor autoantibody
TSH: thyroid-stimulating hormone
WTCCC2: Wellcome Trust case-control consortium
YOGD: young-onset GD

## Additional Information

***Disclosure Summary:*** I certify that neither I nor my coauthors have a conflict of interest as described above that is relevant to the subject matter or materials included in this work.

***Data Availability:*** All data generated or analyzed during this study are included in this published article or in the data repositories listed in References.

## References

[1] Williamson S, Greene SA Incidence of thyrotoxicosis in childhood: a national population based study in the UK and Ireland. Clin Endocrinol (Oxf). 2010;72(3):358-363. 19769613 10.1111/j.1365-2265.2009.03717.x

[2] Nyström HF, Jansson S, Berg G Incidence rate and clinical features of hyperthyroidism in a long-term iodine sufficient area of Sweden (Gothenburg) 2003-2005. Clin Endocrinol (Oxf). 2013;78(5):768-776. 23421407 10.1111/cen.12060

[3] Hussain YS, Hookham JC, Allahabadia A, Balasubramanian SP Epidemiology, management and outcomes of Graves’ disease-real life data. Endocrine. 2017;56(3):568-578. 28478488 10.1007/s12020-017-1306-5 PMC5435772

[4] Rodanaki M, Lodefalk M, Forssell K, Arvidsson CG, Forssberg M, Åman J The Incidence of childhood thyrotoxicosis is increasing in both girls and boys in Sweden. Horm Res Paediatr. 2019;91(3):195-202. 31096231 10.1159/000500265 PMC6690413

[5] Havgaard Kjær R, Smedegård Andersen M, Hansen D Increasing incidence of juvenile thyrotoxicosis in denmark: a nationwide study, 1998-2012. Horm Res Paediatr. 2015;84(2):102-107. 26111962 10.1159/000430985

[6] Flynn RW, MacDonald TM, Morris AD, Jung RT, Leese GP The thyroid epidemiology, audit, and research study: thyroid dysfunction in the general population. J Clin Endocrinol Metab. 2004;89(8):3879-3884. 15292321 10.1210/jc.2003-032089

[7] Abraham P, Avenell A, McGeoch SC, Clark LF, Bevan JS Antithyroid drugregimen for treating Graves’ hyperthyroidism. Cochrane Database Syst Rev. 2010;(1):Cd003420. 10.1002/14651858.CD003420.pub4 PMC6599817 20091544

[8] Rabon S, Burton AM, White PC Graves’ disease in children: long-term outcomes of medical therapy. Clin Endocrinol (Oxf). 2016;85(4):632-635. 27169644 10.1111/cen.13099

[9] Ringold DA, Nicoloff JT, Kesler M, Davis H, Hamilton A, Mack T Further evidence for a strong genetic influence on the development of autoimmune thyroid disease: the California twin study. Thyroid. 2002;12(8):647-653. 12225632 10.1089/105072502760258613

[10] Brix TH, Kyvik KO, Christensen K, Hegedüs L Evidence for a major role of heredity in Graves’ disease: a population-based study of two Danish twin cohorts. J Clin Endocrinol Metab. 2001;86(2):930-934. 11158069 10.1210/jcem.86.2.7242

[11] Pujol-Borrell R, Giménez-Barcons M, Marín-Sánchez A, Colobran R Genetics of Graves’ disease: special focus on the role of TSHR gene. Horm Metab Res. 2015;47(10):753-766. 26361261 10.1055/s-0035-1559646

[12] Chu X, Yang M, Song ZJ, et al. Fine mapping MHC associations in Graves’ disease and its clinical subtypes in Han Chinese. J Med Genet. 2018;55(10):685-692. 29987165 10.1136/jmedgenet-2017-105146 PMC6161647

[13] Ting WH, Chien MN, Lo FS, et al. Association of cytotoxic T-lymphocyte-associated protein 4 (CTLA4) gene polymorphisms with autoimmune thyroid disease in children and adults: case-control study. PLoS One. 2016;11(4):e0154394. 27111218 10.1371/journal.pone.0154394 PMC4844099

[14] Velaga MR, Wilson V, Jennings CE, et al. The codon 620 tryptophan allele of the lymphoid tyrosine phosphatase (LYP) gene is a major determinant of Graves’ disease. J Clin Endocrinol Metab. 2004;89(11):5862-5865. 15531553 10.1210/jc.2004-1108

[15] Chen X, Hu Z, Liu M, et al. Correlation between CTLA-4 and CD40 gene polymorphisms and their interaction in Graves’ disease in a Chinese Han population. BMC Med Genet. 2018;19(1):171. 30223781 10.1186/s12881-018-0665-y PMC6142355

[16] Vos XG, Endert E, Tijssen JG, Wiersinga WM Genotypes in relation to phenotypic appearance and exposure to environmental factors in Graves’ hyperthyroidism. Eur J Endocrinol. 2012;167(6):783-792. 22968483 10.1530/EJE-12-0651

[17] Jurecka-Lubieniecka B, Ploski R, Kula D, et al. Association between age at diagnosis of Graves’ disease and variants in genes involved in immune response. PLoS One. 2013;8(3):e59349. 23544060 10.1371/journal.pone.0059349 PMC3609789

[18] Skórka A, Bednarczuk T, Bar-Andziak E, Nauman J, Ploski R Lymphoid tyrosine phosphatase (PTPN22/LYP) variant and Graves’ disease in a Polish population: association and gene dose-dependent correlation with age of onset. Clin Endocrinol (Oxf). 2005;62(6):679-682. 15943829 10.1111/j.1365-2265.2005.02279.x

[19] Brown RS, Lombardi A, Hasham A, et al. Genetic analysis in young-age-of-onset Graves’ disease reveals new susceptibility loci. J Clin Endocrinol Metab. 2014;99(7):E1387-E1391. 24684463 10.1210/jc.2013-4358 PMC4079314

[20] Yuan FF, Ye XP, Liu W, et al.; China Consortium for the Genetics of Autoimmune Thyroid Disease Genetic study of early-onset Graves’ disease in the Chinese Han population. Clin Genet. 2018;93(1):103-110. 28598035 10.1111/cge.13072

[21] Medici M, Porcu E, Pistis G, et al. Identification of novel genetic loci associated with thyroid peroxidase antibodies and clinical thyroid disease. PLoS Genet. 2014;10(2):e1004123. 24586183 10.1371/journal.pgen.1004123 PMC3937134

[22] Kuś A, Szymański K, Peeters RP, et al. The association of thyroid peroxidase antibody risk loci with susceptibility to and phenotype of Graves’ disease. Clin Endocrinol (Oxf). 2015;83(4):556-562. 25345847 10.1111/cen.12640

[23] Kuś A, Radziszewski M, Glina A, et al. Paediatric-onset and adult-onset Graves’ disease share multiple genetic risk factors. Clin Endocrinol (Oxf). 2019;90(2):320-327. 30358895 10.1111/cen.13887

[24] Purcell S, Neale B, Todd-Brown K, et al. PLINK: a tool set for whole-genome association and population-based linkage analyses. Am J Hum Genet. 2007;81(3):559-575. 17701901 10.1086/519795 PMC1950838

[25] IBM Corp. Released 2017. *IBM SPSS Statistics for Windows, Version 25.0*. Armonk, NY: IBM Corp.

[26] Review Manager (RevMan) [Computer program]. Version 5.3. Copenhagen: The Nordic Cochrane Centre, The Cochrane Collaboration; 2014 https://training.cochrane.org/online-learning/core-software-cochrane-reviews/revman/revman-5-download. Accessed December 2019.

[27] Dudbridge F Likelihood-based association analysis for nuclear families and unrelated subjects with missing genotype data. Hum Hered. 2008;66(2):87-98. 18382088 10.1159/000119108 PMC2386559

[28] Zerbino DR, Achuthan P, Akanni W, et al. Ensembl 2018. Nucleic Acids Res. 2018;46(D1):D754-D761. 29155950 10.1093/nar/gkx1098 PMC5753206

[29] Tomer Y, Menconi F, Davies TF, et al. Dissecting genetic heterogeneity in autoimmune thyroid diseases by subset analysis. J Autoimmun. 2007;29(2-3):69-77. 17644307 10.1016/j.jaut.2007.05.006

[30] Jurecka-Lubieniecka B, Bednarczuk T, Ploski R, et al. Differences in gene-gene interactions in Graves’ disease patients stratified by age of onset. PLoS One. 2016;11(3):e0150307. 26943356 10.1371/journal.pone.0150307 PMC4778933

[31] Kulski JK Long noncoding RNA HCP5, a hybrid HLA class I endogenous retroviral gene: structure, expression, and disease associations. Cells. 2019;8(5):480. 10.3390/cells8050480 PMC6562477 31137555

[32] Okada Y, Momozawa Y, Ashikawa K, et al. Construction of a population-specific HLA imputation reference panel and its application to Graves’ disease risk in Japanese. Nat Genet. 2015;47(7):798-802. 26029868 10.1038/ng.3310

[33] Burton PR, Clayton DG, Cardon LR, et al. Association scan of 14,500 nonsynonymous SNPs in four diseases identifies autoimmunity variants. Nat Genet. 2007;39(11):1329-1337. 17952073 10.1038/ng.2007.17 PMC2680141

[34] Jacobson EM, Huber A, Tomer Y The HLA gene complex in thyroid autoimmunity: from epidemiology to etiology. J Autoimmun. 2008;30(1-2):58-62. 18178059 10.1016/j.jaut.2007.11.010 PMC2244911

[35] Simmonds MJ, Howson JM, Heward JM, et al. A novel and major association of HLA-C in Graves’ disease that eclipses the classical HLA-DRB1 effect. Hum Mol Genet. 2007;16(18):2149-2153. 17597093 10.1093/hmg/ddm165

[36] Yin X, Sachidanandam R, Morshed S, Latif R, Shi R, Davies TF mRNA-Seq reveals novel molecular mechanisms and a robust fingerprint in Graves’ disease. J Clin Endocrinol Metab. 2014;99(10):E2076-E2083. 24971664 10.1210/jc.2014-1735 PMC4184074

[37] Fujinami RS, von Herrath MG, Christen U, Whitton JL Molecular mimicry, bystander activation, or viral persistence: infections and autoimmune disease. Clin Microbiol Rev. 2006;19(1):80-94. 16418524 10.1128/CMR.19.1.80-94.2006 PMC1360274

[38] Tohkin M, Kaniwa N, Saito Y, et al.; Japan Pharmacogenomics Data Science Consortium A whole-genome association study of major determinants for allopurinol-related Stevens-Johnson syndrome and toxic epidermal necrolysis in Japanese patients. Pharmacogenomics J. 2013;13(1):60-69. 21912425 10.1038/tpj.2011.41

[39] Thørner LW, Erikstrup C, Harritshøj LH, et al. Impact of polymorphisms in the HCP5 and HLA-C, and ZNRD1 genes on HIV viral load. Infect Genet Evol. 2016;41:185-190. 27083073 10.1016/j.meegid.2016.03.037

[40] Ciccacci C, Perricone C, Ceccarelli F, et al. A multilocus genetic study in a cohort of Italian SLE patients confirms the association with STAT4 gene and describes a new association with HCP5 gene. PLoS One. 2014;9(11):e111991. 25369137 10.1371/journal.pone.0111991 PMC4219822

[41] Colafrancesco S, Ciccacci C, Priori R, et al. STAT4, TRAF3IP2, IL10, and HCP5 polymorphisms in Sjögren’s syndrome: association with disease susceptibility and clinical aspects. J Immunol Res. 2019;2019:7682827. 30882006 10.1155/2019/7682827 PMC6387711

[42] Liu Y, Helms C, Liao W, et al. A genome-wide association study of psoriasis and psoriatic arthritis identifies new disease loci. PLoS Genet. 2008;4(3):e1000041. 18369459 10.1371/journal.pgen.1000041 PMC2274885

[43] Hur K, Kim SH, Kim JM Potential implications of long noncoding RNAs in autoimmune diseases. Immune Netw. 2019;19(1):e4. 30838159 10.4110/in.2019.19.e4 PMC6399094

[44] Shirasawa S, Harada H, Furugaki K, et al. SNPs in the promoter of a B cell-specific antisense transcript, SAS-ZFAT, determine susceptibility to autoimmune thyroid disease. Hum Mol Genet. 2004;13(19):2221-2231. 15294872 10.1093/hmg/ddh245

[45] Wu GC, Pan HF, Leng RX, et al. Emerging role of long noncoding RNAs in autoimmune diseases. Autoimmun Rev. 2015;14(9):798-805. 25989481 10.1016/j.autrev.2015.05.004

[46] Martin TC, Illieva KM, Visconti A, et al. Dysregulated antibody, natural killer cell and immune mediator profiles in autoimmune thyroid diseases. Cells. 2020;9:665. 10.3390/cells9030665 PMC7140647 32182948

[47] Christensen NJ, Habekost G, Bratholm P A RNA transcript (Heg) in mononuclear cells is negatively correlated with CD14 mRNA and TSH receptor autoantibodies. Clin Exp Immunol. 2008;154(2):209-215. 18778364 10.1111/j.1365-2249.2008.03744.x PMC2612712

[48] Fagerberg L, Hallström BM, Oksvold P, et al Analysis of the human tissue-specific expression by genome-wide integration of transcriptomics and antibody-based proteomics. Mol Cell Proteomics. 2014;13(2):397-406. 24309898 10.1074/mcp.M113.035600 PMC3916642

[49] Montecino-Rodriguez E, Berent-Maoz B, Dorshkind K Causes, consequences, and reversal of immune system aging. J Clin Invest. 2013;123(3):958-965. 23454758 10.1172/JCI64096 PMC3582124

[50] Vos XG, Endert E, Zwinderman AH, Tijssen JG, Wiersinga WM Predicting the risk of recurrence before the start of antithyroid drug therapy in patients with Graves’ hyperthyroidism. J Clin Endocrinol Metab. 2016;101(4):1381-1389. 26863422 10.1210/jc.2015-3644

